# The Tobacco β-Cembrenediol: A Prostate Cancer Recurrence Suppressor Lead and Prospective Scaffold via Modulation of Indoleamine 2,3-Dioxygenase and Tryptophan Dioxygenase

**DOI:** 10.3390/nu14071505

**Published:** 2022-04-04

**Authors:** Ethar A. Mudhish, Abu Bakar Siddique, Hassan Y. Ebrahim, Khaldoun S. Abdelwahed, Judy Ann King, Khalid A. El Sayed

**Affiliations:** 1 School of Basic Pharmaceutical and Toxicological Sciences, College of Pharmacy, University of Louisiana at Monroe, 1800 Bienville Drive, Monroe, LA 71201, USA; mudhishea@warhawks.ulm.edu (E.A.M.); siddique@ulm.edu (A.B.S.); ebrahim@ulm.edu (H.Y.E.); abdelwks@warhawks.ulm.edu (K.S.A.); 2 Department of Pathology and Translational Pathobiology, LSU Health Shreveport, 1501 Kings Highway, Shreveport, LA 71103, USA; judy.king@lsuhs.edu

**Keywords:** (4*R*)-cembratriene-4,6-diol, IDO1 and TDO2, nude mice, prostate cancer, tobacco cembranoids, tobacco leaves, tumor recurrence

## Abstract

Prostate cancer (PC) is the second leading cause of death in men in the US. PC has a high recurrence rate, and limited therapeutic options are available to prevent disease recurrence. The tryptophan-degrading enzymes 2,3-indoleamine dioxygenase (IDO1) and tryptophan dioxygenase (TDO2) are upregulated in invasive PC. (1*S*,2*E*,4*R*,6*R*,7*E*,11*E*)-2,7,11-cembratriene-4,6-diol (β-CBT) and its C-4 epimer α-CBT are the precursors to key flavor ingredients in tobacco leaves. Nearly 40–60% of β- and α-CBT are purposely degraded during commercial tobacco fermentation. Earlier, β-CBT inhibited invasion, reversed calcitonin-stimulated transepithelial resistance decrease, and induced tighter intercellular barriers in PC-3M cells. This study demonstrates the in vitro β-CBT anti-migratory (wound-healing assay) and anti-clonogenicity (colony-formation assay) activities against five diverse human PC cell lines, including the androgen-independent PC-3, PC-3M, and DU-145, the castration-recurrent CWR-R1ca, and the androgen-dependent CWR-22rv1. Meanwhile, β-CBT potently suppressed in vivo locoregional and distant recurrences after the primary tumor surgical excision of PC-3M-Luc cell tumor engrafted in male nude mice. β-CBT treatments suppressed organ and bone metastasis and lacked any major toxicity over the 60-day study course. β-CBT treatments significantly suppressed IDO1, TDO2, and their final metabolite kynurenine levels in PC-3M cells. β-CBT treatments significantly suppressed the tumor recurrence marker PSA and kynurenine levels in treated animals’ plasma. β-CBT emerges as a promising PC recurrence suppressive lead.

## 1. Introduction

One in every five men is a survivor treated for prostate cancer (PC). Unfortunately, the disease often recurs in 30% of patients within 5–10 years, presenting as an incurable metastatic form [1,2]. Despite progress being made towards androgen-suppression mechanisms, including LH/FSH modulators, anti-androgen receptor small molecules, CYP17A and 27A1 inhibitors, chemotherapy, radiation, and immunotherapy, they lack the curative efficacy, especially in metastatic disease patients, who have a poor overall survival rate [1,2,3]. Thus, it is imperative that new, effective, affordable, and safe interventions are developed to control metastatic PC [1].

**Scheme 1:**
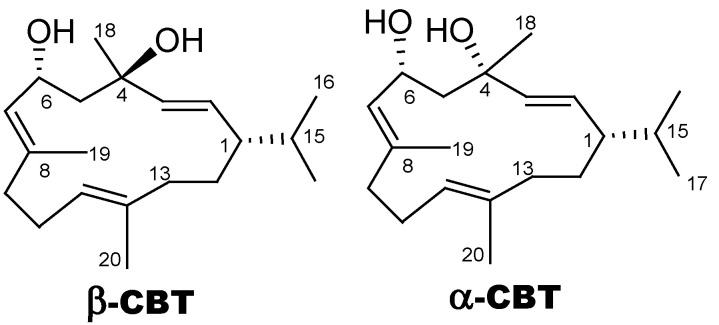
Chemical structure of major tobacco cembranoids.

Cembranoids are natural diterpenes possessing 14-membered macrocyclic rings substituted by an isopropyl residue at C-1 and three symmetrically disposed methyl groups at positions C-4, C-8, and C-12. Cembranoids occur in conifers and tobacco, alligators, termites, ants, and marine soft corals [4,5,6,7]. The earliest discovered natural cembranoid was the (+)-cembrene from pine oleoresin [5,6]. Later, the leaf and flower cuticular wax of most *Nicotiana* species afforded high amounts of the cembranoid diterpenes (1*S*,2*E*,4*R*,6*R*,-7*E*,11*E*)-2,7,11-cembratriene-4,6-diol (β-CBT) and (1*S*,2*E*,4*S*,6*R*,7*E*,11*E*)-2,7,11-cembratriene-4,6-diol (α-CBT) (Scheme 1) [5,6,7]. The structure and absolute stereochemistry of β-CBT was based on X-ray crystallography [5,6]. Both cembranoids are essential flavor ingredients in tobacco [4,5,6,7]. Biodegradation of β-CBT and α-CBT during cure fermentation or flue curing of tobacco leaf leads to their degradation to a range of flavor ingredients with 8–19 carbon skeletons by opening the macrocycle at C-5/C-6 [4,5,6,7]. Tobacco cembranoids showed diverse bioactivities, including behavioral sensitization to nicotine, protective effects against neurodegeneration, reduction in smoking initiation, insecticidal, antimicrobial, prostaglandin and plant growth suppression, in addition to fungal spore germination inhibitory activities [4,5,6,7,8,9,10,11].

Cembranoids β-CBT and α-CBT were identified as anticancer ingredients in cigarette smoke condensates [12]. They inhibited the induction of Epstein–Barr virus (EBV) early antigen by the phorbol ester, 12-*O*-tetradecanoyl-phorbol-13-acetate (TPA), in lymphoblastoid Raji cells and inhibited the ornithine decarboxylase induction activity by TPA in mice epidermis [13,14]. These promising activities were not marred by any observed toxicity. Toxicity study of β-CBT showed a subcutaneous maximum tolerated single dose greater than 98 mg/kg in rats [9]. Meanwhile, α-CBT induced hepatocellular carcinoma apoptosis via modulating the p53-PUMA, PI3K-Akt, and IL-1-NF-κB-IAP pathways [15]. The α-CBT and its semisynthetic analogs were identified as potent inhibitors for triple negative breast cancer (TNBC) cells through targeting its c-MET receptor tyrosine kinase activity [16]. The α-CBT and its hydroxylated mammalian metabolites identified as potent antagonists for VEGFR2, potently suppressing the in vivo angiogenesis of TNBC in a nude mouse xenograft model [17]. The tobacco cembranoid β-CBT and its 4-*O*-methyl ether showed potent anti-invasive activity in spheroid disaggregation assay against the human PC cell line PC-3M cells at sub-μM doses, while it was non-significantly cytotoxic even at 50 μM treatment dose [18,19]. Earlier, β-CBT at 50 nM treatment dose reversed the calcitonin-stimulated reduction in transepithelial resistance and enhanced the paracellular permeability in PC-3M cells [18]. β-CBT was also proven to stimulate tight junction formation between PC-3M cells, producing a tighter intercellular barrier and enhancing the cell–cell adhesion [18]. Concurrently, β-CBT at 10–50 nM potently inhibited the calcitonin-stimulated PC-3M cells invasion in Matrigel assay [18].

Tryptophan is one of the least abundant essential amino acids and humans rely on its dietary intake as the sole supply source [20]. Tryptophan is required for cell growth and considered a critical building precursor for diverse protein and bioactive small-molecule biosynthesis [20]. Most of the free tryptophan catabolism was achieved through the kynurenine (Kyn) pathway, resulting in smaller metabolites, which play important roles in neurotransmission, immunity, and oncogenesis [20]. Tryptophan oxidative catabolism by indoleamine 2,3-dioxygenase (IDO) and/or tryptophan dioxygenase (TDO2) cleave its indole ring, affording *N*-formyl-l-kynurenine before ending up as Kyn.

IDO1 and TDO2 are dysregulated in multiple cancer types [20,21]. Kyn produced by fibroblast induced the formation of E-cadherin-aryl hydrocarbon receptor (AhR)-sphase kinase-associated protein 2 complex, reducing E-cadherin and enhancing breast cancer invasiveness [21]. Notably, the motility of breast cancer co-cultured with fibroblasts producing Kyn was significantly enhanced. This motility enhancement was counteracted and suppressed by the IDO inhibitor 1-methyltryptophan [21]. Furthermore, tryptophan depletion and Kyn upregulation resulted in immunosuppression via natural killer T cell activation and the production of INF-γ, which upregulates IDO1. Therefore, IDO induction provides a negative feedback activity that restrains the T cell activation to sustain host cell integrity, blocking its ability to reject tumors and promoting immune-equivocation [22,23]. The indoleamine dioxygenase-kynurenine -aryl hydrocarbon receptor (IDO–Kyn–AhR) axis was also identified as a driving signaling pathway in thyroid tumorigenesis, mediating an immunosuppressive microenvironment that promoted the acquisition of the mesenchymal phenotype, leading to enhanced tumor invasiveness and metastasis [24]. It is well documented that IDO activity in patients with advanced PC is dysregulated [25]. IDO is upregulated after treatment with DNA vaccine encoding the prostatic acid phosphatase tumor antigen and PD-1 blockade with pembrolizumab [25]. Therefore, IDO inhibition is highly recommended for future anti-PC immunotherapy clinical trials [25]. Recent literature also indicated the ability of the Kyn pathway to activate multiple tumorigenesis pathways, including phospoinositide-3 kinase (PI3K), extracellular signal-regulated kinase (ERK), Wnt/β-catenin, p53, bridging integrator 1 (BIN-1), cyclooxygenase 2 (COX-2), cyclin-dependent kinase (CDK) and collagen type XII α1 chain (COL12A1) [26]. Thus, the dysregulation of IDO and Kyn confer poor prognosis of several human malignancies, including prostate, breast, pancreatic, gastrointestinal, gynecological, and several other cancers [26,27].

This study demonstrates the anti-metastatic and PC recurrence suppressive effects of β-CBT treatment in a heterotopic nude mouse xenograft model. Oral administration of β-CBT at a daily oral dose of 15 mg/kg curbed the locoregional and distant recurrence of PC. β-CBT prevented organ and significantly suppressed bone metastasis and lacked any major toxicity over the 60-day study course. In addition, in vitro data shed light on the ability of β-CBT to control PC cell motility, where β-CBT showed notable anti-migratory and anti-clonogenicity activities against five PC cell lines in wound-healing and colony-formation assay models, respectively, at subtoxic treatment doses without adversely affecting cellular viability. Therefore, β-CBT emerges as an effective and promising PC recurrence and metastasis suppressive lead appropriate for near future development as a prospective anti-PC nutraceutical.

## 2. Materials and Methods

### 2.1. β-CBT Isolation

β-CBT was isolated from fresh Virginia and Oriental tobacco blend purchased from Custom Blends, NY, extracted initially by CH_2_Cl_2_ 3X and later subjected to vacuum liquid chromatography on normal phase Si gel 60 using *n*-hexane-EtOAc gradient mixtures. Preparative HPLC (Cosmosil RP-C-18) was performed using CH_3_CN-H_2_O gradient elution system to afford β-CBT (>95% purity), guided by TLC, NMR, and MS data. β-CBT identity was confirmed by ^1^H NMR using the chemical shift and the multiplicity of its diagnostic methine proton H-6 at 4.81 ppm, resonating as doublet of double doublet (ddd) and H_3_-16 and H_3_-17 isopropyl methyl doublets at δ 0.82, and 0.86, respectively. Furthermore, the ^13^C NMR chemical shift data further confirmed the β-CBT identity via the C-6 methine carbon resonating at δ_C_ 67.5. Thin layer chromatography (TLC): R_f_ value 0.42 (Si gel, *n*-hexane-EtOAc 1:1) [16,17,19,28].

### 2.2. Cell Lines and Culture Conditions

Human PC cell lines PC-3M, PC-3M-Luc, PC-3, and DU-145 were purchased from the American Type Culture Collection (ATCC, Rockville, MD, USA). The CWR-R1ca and CWR-22rv1 cells were kindly provided by Dr. Zakaria Abd Elmageed, Department of Pharmacology, Edward Via College of Osteopathic Medicine, Monroe, LA, USA. All cells were cultured in RPMI-1640 medium supplemented with 10% fetal bovine serum (FBS), amphotericin B (2.5 µg/mL), streptomycin (100 µg/mL), and penicillin G (100 IU/mL) and maintained in a humidified incubator with 5% CO_2_ at 37 °C. For sub-culturing at 70–80% confluency, cells were washed with Ca^2+^- and Mg^2+^-free phosphate-buffered saline (PBS) and detached with 0.05% trypsin containing 0.02% ethylenediamine-tetraacetic acid in PBS for up to 5 min at 37 °C. Complete serum medium was added, and cell pellet was collected by centrifugation for subculturing in fresh medium.

### 2.3. Compound Preparation and Stock Solution

β-CBT was dissolved in dimethyl sulfoxide (DMSO) to provide a stock solution of 25 mM, which used to prepare different concentrations of β-CBT treatment media. DMSO final concentration was maintained fixed in all treatment groups within all given experiments and never exceeded 0.5% of each.

### 2.4. Assesment of β-CBT Effects on PC Cells Viability

β-CBT growth inhibitory effects on a diverse panel of PC cell lines determined by using 3-(4,5-dimethylthiazolyl-2)-2,5-diphenyltetrazolium bromide (MTT) assay. Cells were treated with β-CBT different concentrations (0–100 µM) over 24–72 h as previously described [16,19,29,30,31,32,33].

### 2.5. Wound-Healing Assay

The in vitro wound-healing assay was used to assess directional two-dimensional cell motility. PC-3, PC-3M, CWR-R1ca, CWR-22rv1 and DU-145 cells were plated in sterile flat-bottom 24-well plates and left to form a sub-confluent cell monolayer per well overnight. Wounds were then scratched in each cell monolayer using a 200 µL sterile pipette tip. After removing the media, cells were washed twice with PBS to remove floating cells. Cells were then incubated with 0.5% serum media containing different doses of β-CBT (in triplicates) for 24 h. Media were removed, and cells were washed with cold PBS, fixed with cold methanol and stained with Giemsa. Wound healing was visualized at 0 time and 24 h or until wound closure completed in vehicle control wells. The distance traveled by the cells was assessed by measuring the wound width at time 24 h or at the end of the experiment hours and subtracted from the wound width at the zero time of treatment. The obtained values were expressed as percentage of migration, setting the gap width at the t_0_ as 100%. Each experiment was performed at least three times, and the distance migrated was calculated in three randomly selected fields per each treatment group [16,29,30,31,32].

### 2.6. Colony Formation Assay

The colony formation assay was conducted in 12-well plates (1 × 10^3^ cells per well) for 13 days. Different β-CBT concentrations (1–15 μM) were used over the experiment period with replacing the media every 3 days. After 13 days, the media were removed, and the cells were washed with cold PBS, fixed with cold methanol, and then stained with Giemsa. The number of colonies in each well was counted using countPHICS software [34]. Each experiment was performed in triplicate to ensure the reproducibility and achieve statistical relevance.

### 2.7. Western Blot Analysis

Initially, all PC cells were plated at 1 × 10^6^ cells/100 mm culture plates in RPMI-1640 media with 10% FBS and allowed to adhere overnight [30]. Later, cells were washed with phosphate-buffered saline (PBS) and treated with the respective control or treatment media containing various subtoxic concentrations of β-CBT for 48 h. After treatment, cells were harvested and washed twice with cold PBS, and lysed in radioimmunoprecipitation assay (RIPA) buffer (Qiagen Sciences Inc., Valencia, CA, USA) at 4 °C for 30 min. Lysates were centrifuged for 10 min at 14,000× *g* and supernatants were collected and stored at −80 °C. For animal tissues or tumors, Dulbecco’s phosphate-buffered saline (DPBS) was prepared as 1 mL DPBS supplemented with 10 μL protease inhibitor. Tumor samples were weighted and added in equivalent weights to DPBS (1 mg sample/5 μL DPBS) and homogenized by using ultrasonic homogenizer (Qsonica Sonicator, Newtown, CT, USA). Each homogenate was lysed in RIPA buffer at 4 °C for 30 min. Lysates were centrifuged for 10 min at 14,000× *g* and supernatants were collected and stored at −80 °C. Protein concentration was determined by using the Pierce BCA Protein Assay Kit (Bio-Rad, Hercules, CA, USA). Protein lysates were loaded as cell lysate 20 μg, and tumor lysate 17 μg per well. All protein samples were separated on 10% sodium dodecyl sulfate polyacrylamide gel electrophoresis (SDS-PAGE) gels and transferred to polyvinylidene difluoride membranes. Membranes blocked with 5% bovine serum albumin (BSA) in 10 mM Tris−HCl containing 50 mM NaCl and 0.1% Tween 20, pH 7.4 (TBST). Membranes were incubated with the indicated primary antibodies overnight at 4 °C and washed 5 times with TBST, and then corresponding horseradish peroxidase-conjugated secondary antibodies were used against each primary antibody for 1.5 h at room temperature and finally rinsed with TBST 5 times. Proteins were detected by using ChemiDoc XRS chemiluminescent gel imaging system and analyzed by Image Lab software (Bio-RAD, Hercules, CA, USA). All antibodies were purchased from ProteinTech (Rosemont, IL, USA) and used at a dilution of 1:1000, unless otherwise stated. Visualization of β-tubulin was used to ensure equal sample loading in each lane. Experiments were repeated at least three times. Representative images are presented in figures.

### 2.8. Animal Model and Treatment Mode

Male athymic nude mice (Foxn^1nu^/Foxn^1+^, 5–6 weeks old) were purchased from Envigo (Indianapolis, IN, USA). All animals were acclimated at the University of Louisiana at Monroe (ULM) animal facility and maintained under clean room conditions in sterile filter top cages with Alpha-Dri bedding. Animals were housed on high-efficiency particulate, air-filtered, ventilated racks at 25 °C, 55–65% relative humidity, and a 12 h light/dark cycle. Mice had free access to pelleted rodent chow (No. 7012, Harlan/Teklad, Madison, WI, USA). Animal experiments were approved by the Institutional Animal Care and Use Committee, ULM, protocol # 19NOV-KES-02, and were handled in strict accordance defined by the NIH guidelines. PC-3M-luciferase labeled (PC-3M-Luc) cells were harvested from cell culture and cells’ pellet was resuspended in cold Matrigel. Approximately 2 × 10^6^ cells per 100 µL Matrigel were implanted into the mouse right back flank. Animals were monitored for tumor growth at the injection site for 2–3 weeks. When the average tumor volume reached 200–300 mm^3^, nearly 30 days after xenografting, animals were injected with (+)-luciferin (150 mg/kg, ip, PerkinElmer, Waltham, MA, USA), anesthetized and bioluminescence imaged using PerkinElmer IVIS Lumina Series III (Waltham, MA, USA) imaging system to define in vivo tumor outlines [30]. Tumors were then surgically excised, and animals were then randomized to vehicle control-receiving group and β-CBT-treated group (*n* = 5 per group). Animals received β-CBT or vehicle control orally by using a flexible plastic (2 mm diameter) gavage tube with stainless steel bite protector, 18 gauge, 3.81 cm long. A dose regimen of β-CBT at 15 mg/kg body weight daily for 8 consecutive weeks was used. At the end of the study, animals were anesthetized and bioluminescence whole body images were captured [29]. Animals were then sacrificed and dissected to isolate different organs (brain, heart, lung, liver, kidney, and spleen) and bioluminescence images were recorded for each organ to assess distant metastasis. Photons emitted from luciferase-expressing cells were quantified using the Living Image software program (PerkinElmer, Waltham, MA, USA). Images representing light intensity (blue least intense and red most intense) were generated and quantified as photons/second [29]. Fresh blood was collected from sacrificed animals, plasma was separated, and the blood was kept at −80 °C for subsequent PSA and Kyn analyses.

### 2.9. Aimals Plasma PSA Level

Mice plasma samples were collected from fresh blood samples at the end of the study after animals were sacrificed. Plasma samples were prepared and analyzed for total plasma prostate specific antigen (PSA), following the manufacturer’s protocol, using MYBiosource ELISA kit (MyBiosource, San Diego, CA, USA).

### 2.10. Animals Plasma Kyn Level

The mice plasma samples were prepared from fresh blood samples after sacrifice. Plasma samples were prepared for analysis of the total plasma Kyn, following the manufacturer’s protocol of the Cell BioVision ELISA kit (BioVision Inc, Milpitas, CA, USA). Samples prepared from PC-3M β-CBT-treated cells were analyzed for total Kyn level following the suggested manufacturer procedure.

### 2.11. Statisticcal Analysis

Data were analyzed using GraphPad Prism software version 9.3.1 (La Jolla, CA, USA). Results are presented as mean ± standard deviation (SD). Differences among various treatment and control groups were determined by the unpaired *t*-test and *p*-values were used for statistical significance, where * *p* < 0.05, ** *p* < 0.01, and *** *p* < 0.001.

## 3. Results

### 3.1. Effects of β-CBT on the Viability of Different Human PC Cells

**Figure 1:**
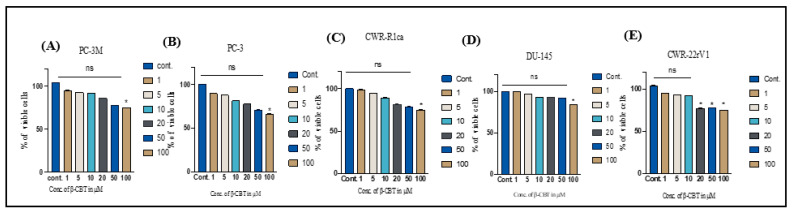
The effects of β-CBT on the viability of a human PC cell lines panel. Bar graphs represent the percent cell viability in vehicle, 1, 5, 10, 20, 50, and 100 µM of β-CBT-treated PC cells. Figures represent treatments to PC-3M (**A**), PC-3 (**B**), CWR-R1ca (**C**), DU-145 (**D**), and CWR-22rv1 (**E**) cells. “ns” indicates statistical non-significance at *p* < 0.05. * indicate statistical significance relative to control cells at *p* < 0.05.

The effects of the β-CBT treatment dose range of 1–100 μM on the viability of the human PC cell lines PC-3M, PC-3, CWR-R1ca, DU-145, and CWR-22rv1 were examined using the MTT experiment. Β-CBT treatments up to 50 μM did not show significant adverse effects on the viability of the five human PC cell lines (Figure 1). This experiment guided the subsequent testing of β-CBT effects against the PC cell line migration and clonogenicity by selecting subtoxic doses of β-CBT at a cut-off limit of 10 μM. Thus, the pooled pharmacological outcomes of the motility-targeting assays were guaranteed to be due to a direct effect and not due to non-selective proliferation or viability suppressive effects. Furthermore, a subtoxic treatment dose of 40 μM β-CBT did not affect the levels of the apoptotic marker caspases 7 and 9 in PC-3M cells, evidenced by Western blot analysis, compared to vehicle-treated control PC-3M cells (Supplementary Figure S1).

### 3.2. β-CBT Reduced the Migration and Colony Formation in Different PC Cells

**Figure 2:**
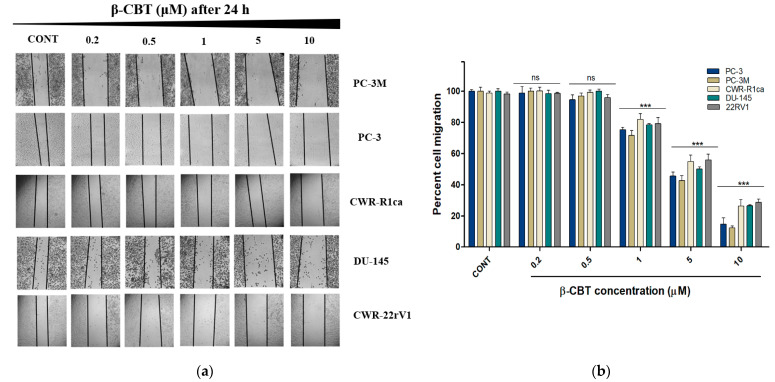
Effects of β-CBT treatments on the migration of PC-3M, PC-3, CWR-R1ca, DU-145, and CWR-22rv1 PC cell lines. (**a**) Representative microscopic photographs of wounds in different PC cell monolayers after 24 h incubation with control and β-CBT treatment at different concentrations (0.2–10 µM). (**b**) Dose–response of different β-CBT concentrations on percent cell migration of treated PC cell lines. Bar graphs represent mean percent cell migration (±SD) at the indicated β-CBT concentration relative to vehicle-treated control cells (CONT, 100% migration). *** indicates statistical significance relative to the control cells at *p* < 0.001, and “ns” indicates statistical non-significance at *p* < 0.05.

**Figure 3:**
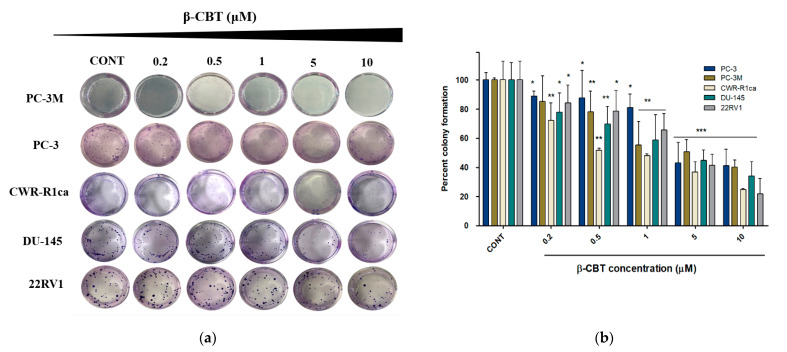
Effects of β-CBT treatments on the clonogenicity of PC-3M, PC-3, CWR-R1ca, DU-145 and CWR-22rv1 PC cells. (**a**) Representative images of PC colonies in control cells, and cell treated with β-CBT treatments for 13 days and stained with Giemsa at the end of the experiment. (**b**) Dose–response of different β-CBT concentrations and percent colony formation. Bar graphs represent the mean percent colony formation (±SD) at indicated concentrations (µM) relative to vehicle-treated control cells (CONT, 100% clonogenicity). *, **, *** indicate statistical significance relative to control cells at *p* < 0.05, *p* < 0.01, *p* < 0.001, respectively.

The effect of β-CBT treatments on the migration of the PC cell lines PC-3M and PC-3, as well as the cell lines CWR-R1ca, DU-145, and CWR-22rv1, was evaluated using the wound-healing assay (WHA). Tumor cells not only have the potential to proliferate, but they also dynamically tend to invade and migrate to the surrounding and even distant locations. Tumor cell migration is a crucial step to the in vivo metastatic cascade. Hence, the WHA was performed to assess the in vitro antimigratory effect of β-CBT treatments against the PC-3M, PC-3, CWR-R1ca, DU-145 and CWR-22rv1 cells. Treatments with β-CBT at the subtoxic concentrations 0.2, 0.5, 1, 5, and 10 μM for 24 h significantly inhibited the migration of all the PC cell lines in a dose-dependent manner, with IC_50_ values (±SD) of 4.5 ± 0.7, 7.3 ± 0.9, 9.8 ± 1.1, 5.3 ± 0.8, and 9.4 ± 0.8 μM for PC-3M, PC-3, CWR-R1ca, DU-145, and CWR-22rv1, respectively (Figure 2 and Table 1). The colony formation assay is an in vitro experiment that closely mimics the in vivo clonogenicity of the disseminated tumor cells that escaped the surgery and/or resisted the therapeutic regimen. These cells will have to form colonies first at the distant site before subsequent pathogenesis [27,35,36]. Different β-CBT treatments in the range of 0.2–10 μM were used to test its ability to inhibit the clonogenicity of PC-3M, PC-3, CWR-R1ca, DU-145, and CWR-22rv1 cells. Β-CBT treatments potently inhibited the colony formation at IC_50_ values (±SD) of 0.94 ± 0.1, 2.93 ± 0.5, 0.34 ± 0.1, 1.21 ± 0.2, and 4.31 ± 0.6 μM for PC-3M, PC-3, CWR-R1ca, DU-145, and CWR-22rv1, respectively (Figure 3 and Table 1).

### 3.3. β-CBT Reduced IDO1 and TDO2 Expression In Vitro in PC-3M Cell Line

**Figure 4:**
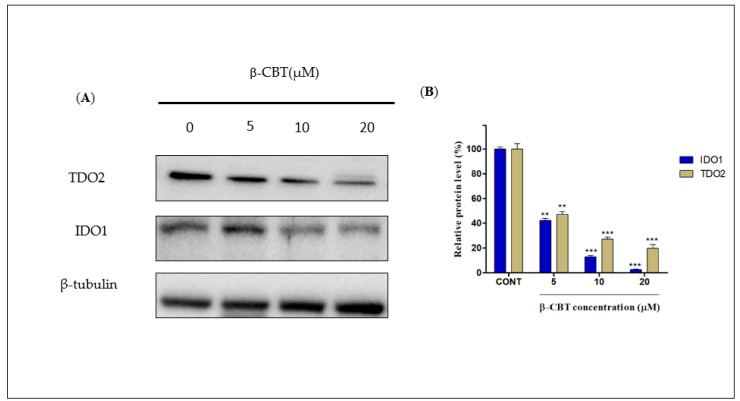
Effect of β-CBT treatments on the expression levels of IDO1 and TDO2 in PC-3M prostate cancer cell lines. (**A**) Immunoblots of IDO1 and TDO2 in cells treated with the vehicle control and 5, 10, and 20 μM β-CBT over 24 h. (**B**) Densitometric analysis of IDO1 and TDO2 expressions in PC-3M cells. Scanning densitometry was obtained for all blots, carried out in triplicate, and the integrated optical density of each band was normalized to β-tubulin in the same blot. Bar graphs represent mean relative protein expression percent (±SD). ** *p* < 0.01 and *** *p* < 0.001 for statistical significance vs. respective vehicle-treated controls.

The heme-containing enzymes IDO1 and TDO2 catalyze the catabolic oxidation of L-tryptophan in humans [20]. IDO1/TDO2 play important roles in cancer cell motility and their immune escape by catalyzing the initial and rate-limiting step of the tryptophan/kynurenine pathway, with its intermediates and end metabolites interfering with natural killer T cell interferon production [20,21,22,23,24,25]. Overexpression of IDO1/TDO2 is also associated with poor prognosis in various cancers [20,21,22,23,24,25]. Recently, the notion of modulating IDO1/TDO2 by small-molecule inhibitors is being assessed in clinical trials testing new PC vaccines [25]. In this piece of work, the in vitro effect of β-CBT on the expression levels of IDO1 and TDO2 in the PC-3M cell line was studied. PC-3M cells were treated with various subtoxic doses of β-CBT (5, 10, and 20 μM) for 24 h, followed by subsequent Western blot analyses of the IDO1 and TDO2 levels. The β-CBT treatments significantly suppressed the IDO1 level in PC-3M cells in a dose-dependent manner, compared to the vehicle-treated control (Figure 4). The IDO1 expression level values were 42.3, 13, and 2.8% for β-CBT treatments of 5, 10, and 20 μM, respectively, in comparison to 100% expression level for the vehicle control-treated PC-3M cells. The TDO2 level also significantly downregulated along with the IDO1 reduction, compared to the vehicle-treated control cells. The TDO2 expression level values were 47.1, 27.2, and 19.8% with β-CBT treatments of 5, 10, and 20 μM, respectively, in comparison to 100% in PC-3M cells treated with vehicle control (Figure 4). Thus, the PC-3M cell line selected for the in vivo study because it is a hormone-independent cell line, has a higher metastatic potential compared to other PC cell lines, and has dysregulated IDO1 and TDO2 expression levels (Figure 4).

### 3.4. β-CBT Inhibited PC-3M-Cells Locoregional Recurrences after Primary Tumor Surgical Excision and Tumor Distant Recurrence

**Figure 5:**
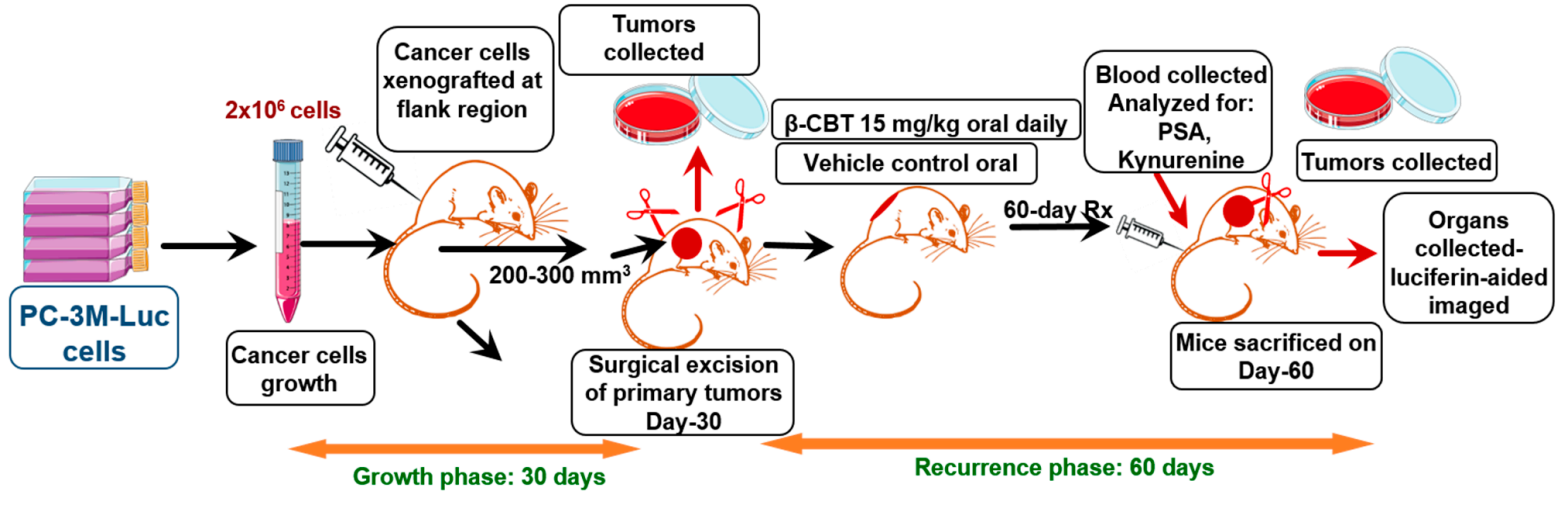
Overview of the animal study experiment.

**Figure 6:**
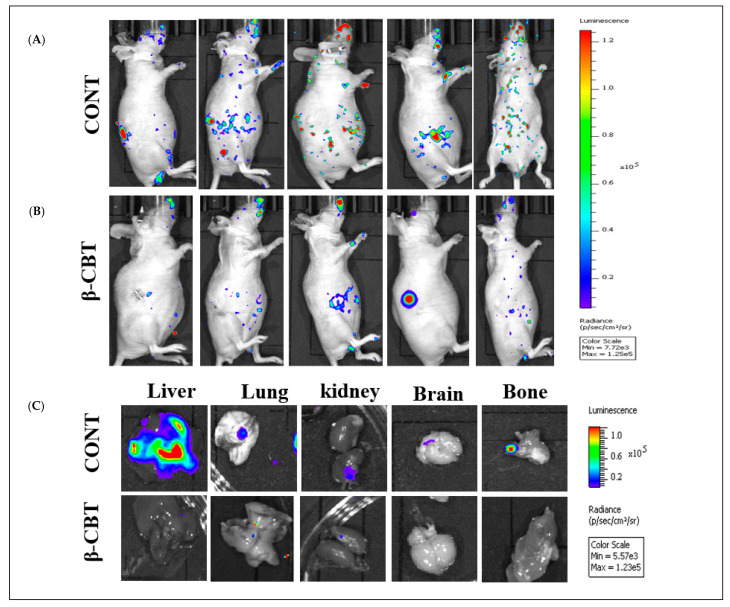

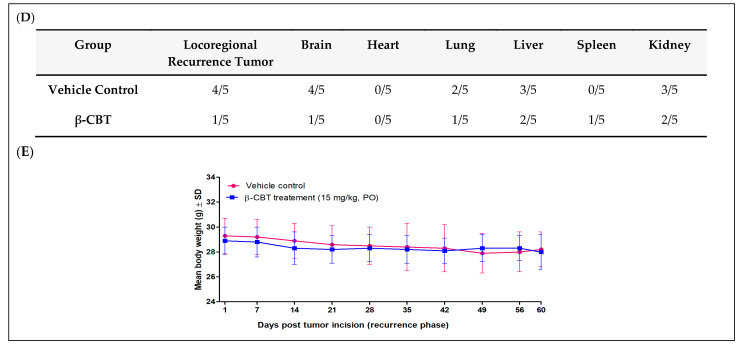
Comparison of the effects of daily oral 15 mg/kg oral β-CBT for 60 days on PC-3M PC cell locoregional and distant recurrence. (**A**) Bioluminescence intensity monitoring of PC-3M-Luc cells in intact animal shows the oral effect of vehicle control and (**B**) β-CBT 15 mg/kg daily oral treatments at the end of experiment course (60 days). (**C**) Bioluminescence comparison of animal organs for β-CBT-treated versus vehicle control-treated groups collected at the study end. (**D**) Number of metastatic organs in β-CBT-treated group versus vehicle control group. (**E**) Comparative monitoring of the effects of β-CBT versus vehicle control treatments on the mean mice total body weight over the recurrence-phase course (60 days). Data points represent mean body weight per group in g ± SD. The mean body weights comparison between vehicle-treated control and β-CBT-treated groups was statistically non-significant, *p* < 0.05.

Recurrence represents a major therapeutic challenge to oncologists, being the leading cause of death in cancer patients. The PC metastatic phenotypes have a significantly higher recurrence rate than other PC phenotypes [27,35,36]. One of the possible recurrence triggers is the activation of dormant-tumor cells. Cellular dormancy is a mode of inactivity that occurs when a temporary mitotic is arrested representing a critical phenomenon of latency [36]. This leads to imparting the metastatic cancer cell refractory to targeted and conventional chemotherapies [36]. To investigate the potential of β-CBT as a PC recurrence inhibitor, a daily oral 15 mg/kg β-CBT treatments continued for an additional 60 days after the primary tumor resection as summarized in the study design overview (Figure 5). Five out of a total of ten mice in vehicle and β-CBT-treated groups developed locoregional recurrence tumors (Figure 6). In the vehicle-treated group, four mice showed both locoregional and distant recurrences evidenced by bioluminescence imaging taken at the end of experiment. Vehicle-treated mice recurred tumors within 19–28 days post-primary tumor excision surgery. Meanwhile, a single β-CBT-treated mouse recurred a very small locoregional tumor around the 35th day post primary tumor surgical excision (Figure 6), suggesting that β-CBT significantly suppressed the development, extended the latency, and delayed the onset of the locoregional PC-3M cell tumor recurrence.

Only one brain, one spleen, two lungs, and two kidneys’ distant recurrence was observed in the β-CBT-treated mouse organs, unlike the vehicle-control-treated mice, which showed distant recurrence/metastatic foci at four hearts, two lungs, three livers, and two kidneys (Figure 6). Over the 60 days of 15 mg/kg β-CBT daily oral therapeutic use, no gross adverse or behavioral responses in the male athymic nude mice were observed. Non-significant changes were observed on the animal total body weight, suggesting potential preliminary β-CBT long-term gross safety (Figure 6).

### 3.5. β-CBT Reduced Mice Plasma Total Prostate-Specific Antigen (PSA)

**Figure 7:**
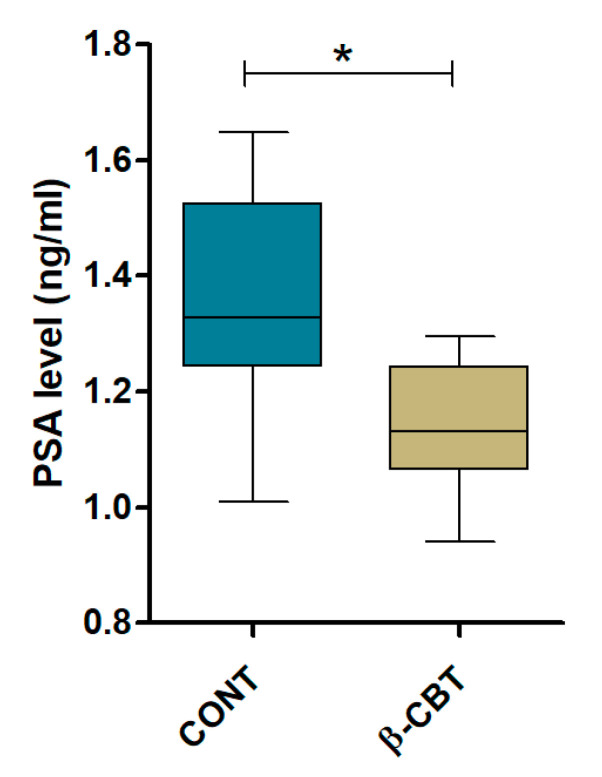
Effect of daily oral 15 mg/kg β-CBT treatments on PSA plasma level in mice xenografted with PC-3M-Luc cells at the study end. Data represent median, minimum and maximum of vehicle-treated control group and β-CBT-treated group. * indicates statistical significance at *p* < 0.05.

β-CBT treatments particularly reduced the prostate-specific antigen (PSA) in blood samples collected from mice after sacrificing with an average PSA level of 1.16 ng/mL in β-CBT-treated mice compared to 1.33 ng/mL in vehicle control-treated mice. This result provides additional evidence for the β-CBT potential to reduce the PC recurrence incidence by oral treatments (Figure 7).

### 3.6. β-CBT Reduced Mice Plasma Kynurenine Level

**Figure 8:**
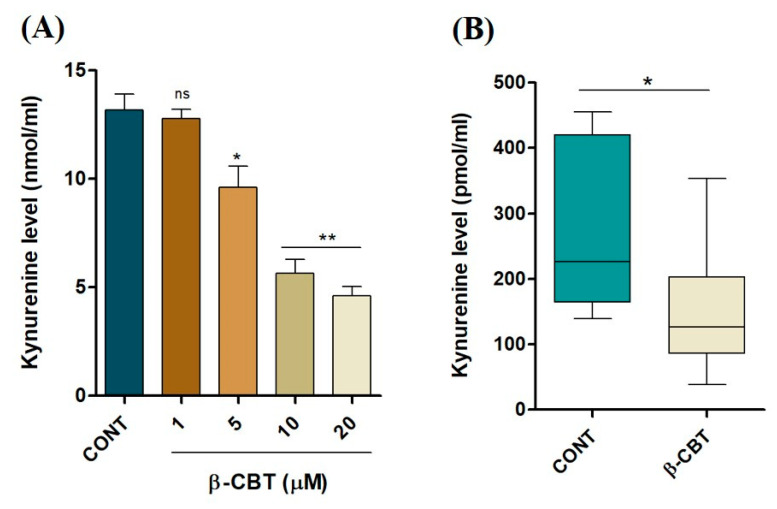
Effect of β-CBT on Kyn level in PC-3M in cell culture and in nude mice plasma xenografted with PC-3M-Luc cells. (**A**) Dose–response effects of the β-CBT treatments on the level of Kyn in in vitro cultured PC-3M cells. Bar graphs represent the mean Kyn level (±SD) at indicated concentrations. (**B**) Comparison of Kyn level in vehicle-treated control versus β-CBT-treated mice plasma. Data represent median, minimum and maximum values. * indicates statistical significance at *p* < 0.05 and ** at *p* < 0.01.

β-CBT treatments notably reduced the mice plasma Kyn level, with a median of 216.1 pmol/mL in β-CBT-treated mice compared to 124.5 pmol/mL in vehicle control-treated mice, which represents a 42.4% reduction in the Kyn plasma level. In addition, Kyn cellular level was assessed in PC-3M cells treated with β-CBT at 1, 5, 10, and 20 μM. The results revealed a dose-dependent decrease in cellular Kyn concentration, with a calculated IC_50_ value of 6.14 µM (Figure 8). Results also highlight the significant Kyn level in vitro in cell cultures in which the Kyn level was in ng/mL, unlike the much lower in vivo plasma Kyn level, which was in pmol/mL level.

## 4. Discussion

Prostate cancer represents a salient health threat to men in the US and worldwide. Although several PC therapeutic options are available, effective recurrence inhibitors are lacking. Current therapies have limited cancer recurrence preventive activity and fail to eradicate residual quiescent tumor cells [27,35]. PC treatment delay can also raise the disease recurrence risk [27,36]. PC survivors have to worry about the disease relapse because recurred tumors usually have an aggressive phenotype with a high morbidity incidence [36].

Nature has been and will continue to be the topmost source for novel drug entities. The high complexity, regioselectivity, and innovative chemistry of natural products are attributed to their source unique biosynthetic enzyme machinery [37,38,39,40]. Natural products have several advantages, including sustained supply source, high novelty, cost-effectiveness, target novelty, selectivity, and relative safety profiles [37,38,39]. Natural products have a standing success record in the oncology arena since more than 50% of today’s anticancer therapeutics are natural products and/or based on or inspired by natural product parents [37].

Cembranoids are a group of natural diterpenoid secondary metabolites produced by plants of *Nicotiana* species. Cembranoids showed several biological potentials mostly in two directions, the neuroprotection and anticancer [41]. Unfortunately, these pharmaceutically useful ingredients are purposely degraded during the commercial tobacco fermentation to produce distinct flavors.

This study builds on our previous report that validated the exceptional potent in vitro anti-PC invasive activity for one of the major tobacco cembranoids, β-CBT, against the androgen-independent PC cell line PC-3M [18]. β-CBT lacked cytotoxicity up to 50 µM, but 50 nM treatment reversed the calcitonin-stimulated oncogenic and invasiveness effects and tightened the intercellular junctions in PC-3M cells [18]. Thus, β-CBT was tested in this study for viability, migratory and clonogenicity suppression activities against a diverse panel of five human PC cell lines. The list included the androgen-independent PC-3M, PC-3, and DU-145 cells, the metastatic castration-resistant-recurrent CWR-R1ca cells, and the androgen-dependent CWR-22rv1cell line. A treatment dose range of 1–100 µM β-CBT did not significantly suppress the viability of the five PC cell lines up to 50 µM in MTT assay (Figure 1). This piece of datum was further validated in PC-3M cell lysates by the Western blot analysis of two main conserved apoptotic markers, caspase 9 and caspase 7 [42]. In principle, caspase 9 is an initiator member of the apoptotic cascade that is autoactivated by various cell death stimuli. Meanwhile, caspase 7 is an executioner protease that cleaves a large set of cellular substrates upon activation by caspase 9. Herein, the results show that treatment of PC-3M cells with 40 µM of β-CBT did not induce significant upregulation of both apoptotic caspases (Supplementary Figure S1). This truly validates the preliminary MTT viability data and would inevitably gauge the β-CBT treatment concentrations in subsequent migratory and colorogenic assays [42].

β-CBT treatments showed good anti-migratory activity in WHA against most of the five PC cell lines, with an IC_50_ range of 1–10 µM over a 24 h treatment period (Figure 2). Colony formation assay is the most relevant in vitro model to distant recurrence, since circulating tumor cells have to adhere and form colonies at the distant organs before subsequent pathogenesis [36]. β-CBT treatments showed good clonogenicity-suppressing activity in colony formation assay against most of the five PC cell lines, with an IC_50_ range of 1–10 µM over 5–14 days of treatment periods (Figure 3). One of the topmost cell lines that showed sensitivity to β-CBT treatments in the migration and clonogenicity assays was PC-3M. Thus, this cell line was selected for a subsequent recurrence suppressive activity study in a nude mice xenograft model.

Indoleamine 2,3-dioxygenase 1 (IDO1) and tryptophan 2,3-dioxygenase 2 (TDO2) play pivotal roles in cancer viability and motility, and they facilitate tumor cells escaping the host immune system by catalyzing the initial step of the Kyn production. Traditionally, the immunosuppressive effect of IDO1 and TDO2 has been attributed to reducing tryptophan level via catalyzing the commitment production step of the kynurenine (Kyn) metabolic pathway. Kyn deregulation is significantly correlated with cancer progression and enhanced tumor motility [21]. Overexpression of IDO1 is considered a negative prognostic marker in several cancer types, including PC [26]. Kyn activates several tumor cell-critical pathways, such as PI3K, ERK, Wnt/β-catenin, COX-2, CDK and others, which can be crucial to tumorigenicity and recurrence [26]. Herein, the Western blotting results suggest that β-CBT can modulate intracellular IDO1/TDO2 in PC-3M cells. Treated PC-3M cells with 5, 10, and 20 μM β-CBT over 24 h showed a significant reduction in IDO1 and TDO2 levels in a dose-dependent manner (Figure 4). The least tested treatment concentration, 5 μM, effectively reduced both IDO1 and TDO2 expression levels in PC-3M cells versus vehicle control treatment (Figure 4). Higher β-CBT treatments, 10 and 20 μM, nearly completely abolished all IDO1 level and reduced more than 80% of TDO2 level (Figure 4). This clearly highlights the potential of β-CBT as a novel small-molecule inhibitor of IDO1 and TDO2. Primary PC surgical excision does not eliminate the potential of local, regional, and/or distant disease relapse completely [3,27,35,43]. Introducing β-CBT as a first-in-class dual inhibitor of IDO1 and DTO2 is expected to warrant future consideration of the IDO1/TDO2 axis as a novel valid molecular target to reduce the risk of PC recurrence and, therefore, improve long-term PC patients’ survival rate. Undoubtedly, a readily available orally active small-molecule IDO1/TDO2 inhibitor for PC recurrence suppression would be more convenient and cost-effective. The β-CBT cost-effectiveness is based on its sustained isolation supply source, and anticipated ease for commercial productions from tobacco flowers, which are not used in commercial tobacco products but also rich in β-CBT.

The currently available PC treatments rely on androgen deprivation via inhibiting androgen synthesis that activates the AR ligand binding domain. The initial response to androgen deprivation therapy is favorable. However, many patients inevitably relapse to a more aggressive and resistant disease form. Currently, several therapeutic options are available for testosterone-dependent PC; however, cancer recurrence inhibitors are lacking. Neoadjuvant and adjuvant therapies fail to eliminate residual dormant tumor cells. Therefore, they have limited effects against cancer recurrence [3,35,36,43]. Thus, there is a dire need for the discovery of novel PC recurrence inhibitors for use by PC survivors to extend their disease-free survival.

We recently validated a new model to assess the recurrence-suppressing activity of a tested compound in nude mice by heterotopic xenografting of tumor cells, allowing the primary tumors to grow to optimal, palpable, detectable size, usually 200–300 mm^3^, then surgically excise the formed primary tumor and begin the tested compound dosing to assess its ability to prevent or suppress locoregional and/or distant tumor recurrences (Figure 5) [29,30,31,32,33]. This tumor volume end point is optimal for successful primary tumor excision surgery as it minimizes animal loss during and/or after the surgery. The results of the present study validate the β-CBT oral activity in experimental animals to suppress the PC recurrence in athymic male nude mice. A 60-day oral daily dosing course with 15 mg/kg β-CBT significantly suppressed PC-3M-Luc locoregional and distant recurrences in athymic nude mice as shown in the bioluminescence images (Figure 6). The few mice treated with β-CBT that showed minor tumor recurrence had extended latency and 12-day tumor recurrence onset delay. It has been documented that 2.6 adult mouse life days are equivalent to a single human year [31,44]. Thus, the 12-day tumor recurrence onset latency in mice produced by β-CBT treatments can be translated to 4.6 years of tumor-free survival in humans. Moreover, β-CBT suppressed the locoregional recurrence of tumor clusters and inhibited the distant recurrence of PC-3M-Luc cells to the brain, lung, liver, kidney, and other organs compared in the vehicle control group (Figure 6).

β-CBT recurrence suppressive effects were not associated with adverse effects on mean mice body weight over the experiment course as compared to the vehicle control group (Figure 6). The high safety profile of β-CBT was further supported by showing no histopathological abnormalities in the H&E-stained liver and heart sections of treated animals versus the vehicle control group (Supplementary Figure S2). Organs from male nude mice (vehicle control and β-CBT 15 mg/kg) were collected at the study end (after surgical excision of primary tumors and continued β-CBT treatment for 60 days). Each organ tissue was fixed in formalin, embedded in paraffin, and H&E-stained sections were prepared. Multiple organs, including liver and heart, were examined histologically for any evidence of toxicity and tumor metastasis. No significant microvesicular/macrovesicular steatosis, inflammation, necrosis, drug crystals, cytoplasmic inclusions, or cholestasis were identified, suggesting no evidence of toxicity in the liver. No significant myocardial inflammation, necrosis, infarction, or fibrosis were observed, indicating no evidence of cardiotoxicity (Supplementary Figure S2).

This study findings suggest the β-CBT potentiality to exhibit potent recurrence suppressive effects without perceptible toxicity, unlike most available cancer therapies. The 60-day use of β-CBT treatments in mice can be comparable to nearly 23 years of use in humans [44], suggesting a high safety profile and highlighting its future potential for application as a prospective nutraceutical for PC survivors. However, additional comprehensive toxicological studies might be warranted.

The prostate-specific antigen PSA is a reliable tumor marker for monitoring post-operative risk of cancer recurrence and considered to be an early detection antigen of PC recurrence [43,45,46,47]. Thus, we evaluated the level of PSA in the collected animal plasma samples at the end of the study as a marker for successful recurrence suppression. The results show that the oral β-CBT treatments optimally reduced the PSA level in mice plasma to 1.16 ng/mL versus 1.33 ng/mL in the vehicle-treated control group (Figure 7). This result provides additional evidence for the β-CBT potential to reduce the PC recurrence incidence by daily oral intake.

Kynurenine (Kyn) is the main rate-limiting metabolic product of tryptophan, the natural substrate to IDO1/TDO2 in the evolutionarily conserved tryptophan/Kyn pathway [20]. The dramatic increase in tryptophan catabolism and Kyn overproduction notably contributes to PC tumorigenicity, progression, and motility [20,21,22,23]. In vitro, β-CBT-treated PC-3M cells at various concentrations over 24 h showed a significant dose-dependent reduction in Kyn level with a calculated IC_50_ value of 6.14 µM (Figure 8). This promising in vitro bioactivity was also validated by measuring the plasma Kyn level in β-CBT-treated mice in comparison to its level in vehicle control-treated mice. The daily oral β-CBT administration demonstrated a considerable reduction in plasma Kyn level with 42.4% calculated inhibition compared to the vehicle control-treated animals (Figure 8). Such piece of datum further validated the in vivo inhibition of IDO1 and TDO2 and can be correlated, at least in-part, to the observable recurrence and metastatic regression in the PC-3M-Luc nude mouse xenograft model. Collectively, the acquired results validate β-CBT as a small-molecule natural product lead suitable for the control of hormone-independent PC recurrence.

## 5. Conclusions

In summary, the current study introduces a small-molecule natural product, β-CBT, which dually reduced IDO1 and TDO2 expressions, reducing the tryptophan catabolic product Kyn, suppressing the in vitro and in vivo motility and clonogenicity of the hormone-independent PC-3M PC cells. β-CBT concomitantly reduced mice plasma levels of Kyn and the PC recurrence marker PSA. It also potently inhibited the locoregional PC-3M-Luc PC cell recurrence after primary tumor surgical excision. The reduction in tumor recurrence was also associated with the downregulation of IDO1/TDO2 levels. Targeting IDO1/TDO2 expression is a novel strategy to control hormone-independent PC recurrence and metastasis. Although β-CBT showed modest in vitro cytotoxic effects on tumor cells at relatively high doses, it induced effective tumor recurrence inhibition in vivo. β-CBT extended treatment use was associated with a preliminary acceptable safety profile, which qualifies this unique natural product to become a prospective nutraceutical for use by PC survivors to extend their disease-free survival. Worth noting, this study is not intended to promote commercial tobacco smoking because it will never change the fact that tobacco smoke and its combustion products possess tobacco-specific carcinogens, which induce several malignancies. In fact, commercial tobacco is low in tobacco cembranoids, including β-CBT, and therefore its use will not prevent PC recurrence. Commercial tobacco fermentations purposely degrade β-CBT to make specific commercial flavors. This study is an effort to promote the raw/fresh tobacco, the economically relevant agricultural crop, for useful pharmaceutical and therapeutic applications.

## Figures and Tables

**Table 1:** Calculated β-CBT IC_50_ values (µM ± SD) in wound-healing and colony-forming assays using multiple human PC cells.

	Prostate Cancer Cell Lines				
	PC-3M	PC-3	CWR-R1ca	DU-145	CWR-22rv1
Wound healing assay	4.50 ± 0.7	7.30 ± 0.9	9.80 ± 1.1	5.30 ± 0.8	9.40 ± 0.8
Colony formation assay	0.49 ± 0.1	2.93 ± 0.5	0.34 ± 0.1	1.21 ± 0.2	4.31 ± 0.6

## Data Availability

Not applicable.

## Supplementary Materials

The following supporting information can be downloaded at: https://www.mdpi.com/article/10.3390/nu14071505/s1, Figure S1: Effects of 40 μM β-CBT versus vehicle control treatments on apoptosis marker caspases 7 and 9. Figure S2: Comparison of the histopathological effects of oral β-CBT versus vehicle control treatments on the nude mouse livers and hearts.
