# Supplementary material for: The Tobacco β-Cembrenediol: A Prostate Cancer Recurrence Suppressor Lead and Prospective Scaffold via Modulation of Indoleamine 2,3-Dioxygenase and Tryptophan Dioxygenase
Source: Nutrients. 2022 Apr 4;14(7):1505. doi: 10.3390/nu14071505 (PMC9003379; doi:10.3390/nu14071505)
Supplement: Supplementary file 1 [file nutrients-14-01505-s001.zip › nutrients-1639875-supplementary.pdf]

nutrients

MDPI

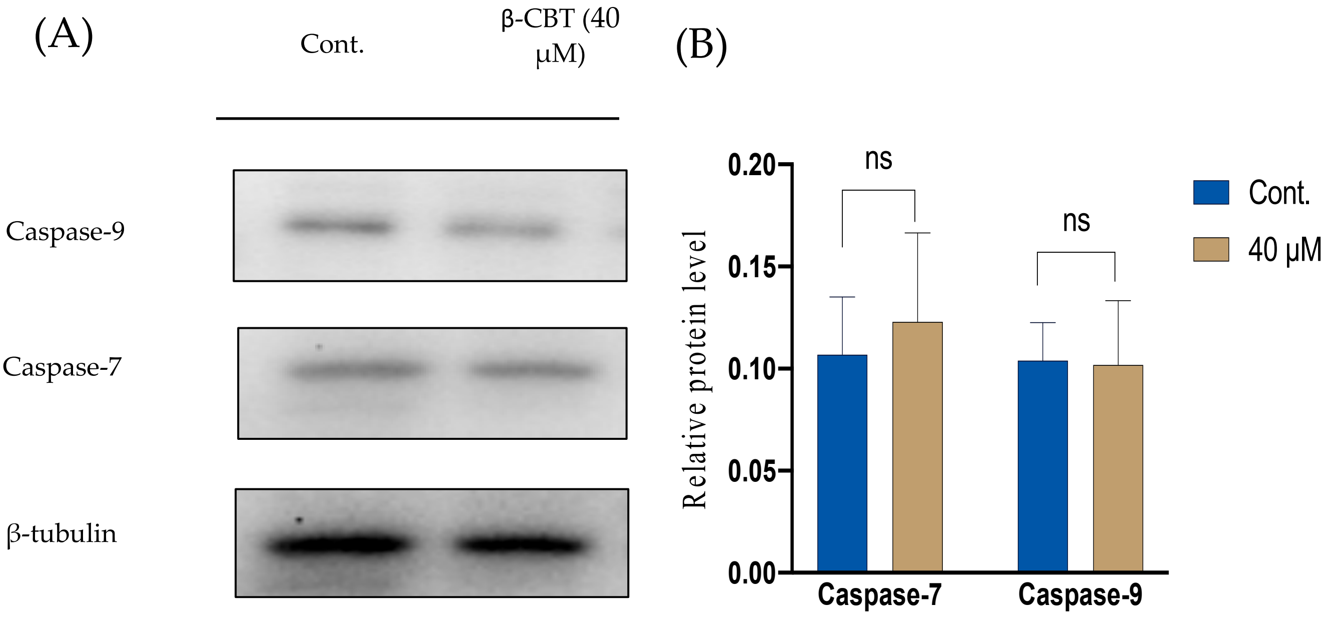

Supplementary Figure S1: Effects of 40 μM β-CBT versus vehicle control treatments on protein level. Western blot showed no significant elevation of apoptosis markers caspase-7 and caspase -9 neither necrosis marker FAS on PC-3M cells treated with 40 μM β-CBT.

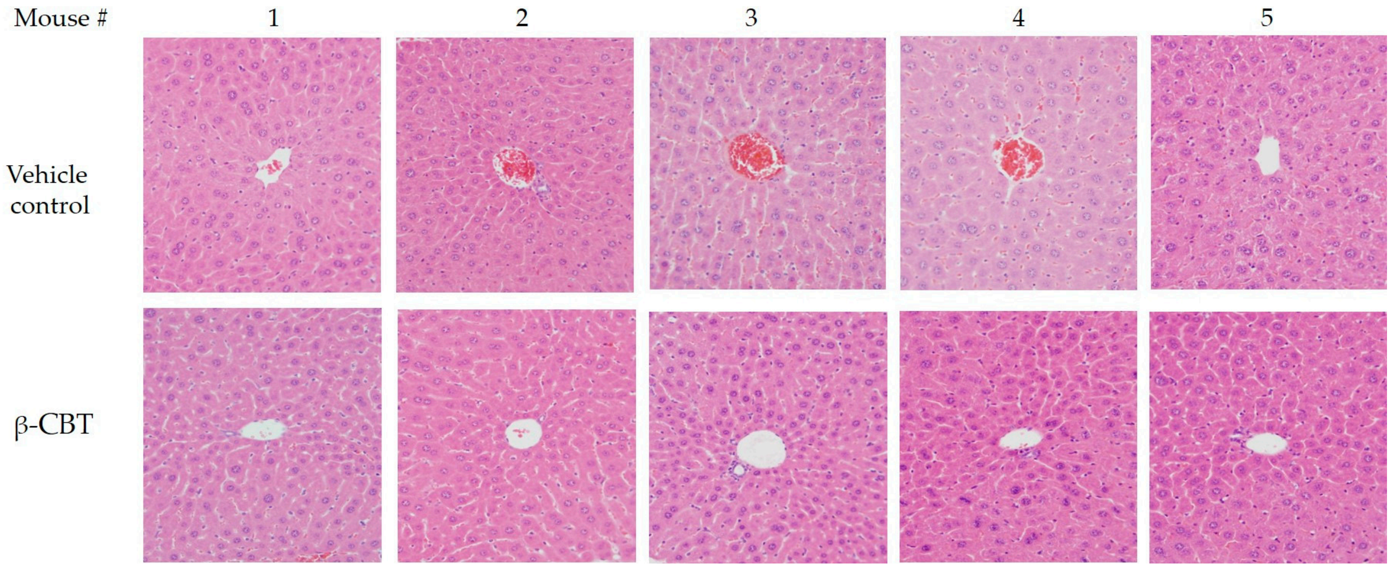

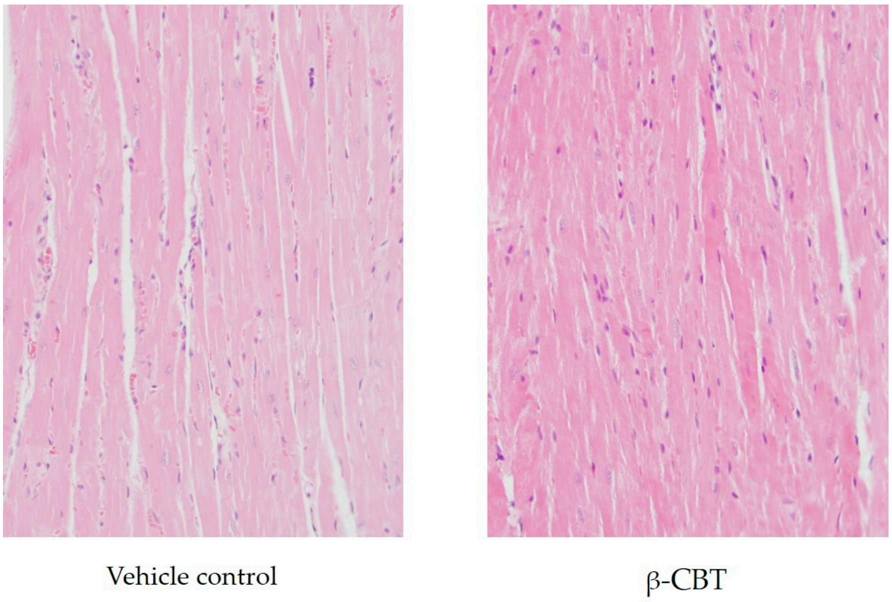

Supplementary Figure S2: Comparison of the histopathological effects of oral β-CBT (15 mg/kg) versus vehicle control treatments on the nude mouse hearts. 20x objective, 200x total magnification.
